# Bioactive Molecules of Microalgae *Haematococcus pluvialis*–Mediated Synthesized Silver Nanoparticles: Antioxidant, Antimicrobial, Antibiofilm, Hemolysis Assay, and Anticancer

**DOI:** 10.1155/bca/8876478

**Published:** 2025-05-02

**Authors:** Yoo-Na Jeon, Su-Ji Ryu, Anbazhagan Sathiyaseelan, Jong-Suep Baek

**Affiliations:** ^1^Department of Bio-Health Convergence, Kangwon National University, Chuncheon 24341, Republic of Korea; ^2^BeNatureBioLab, Chuncheon 24206, Republic of Korea

**Keywords:** antibacterial, anticancer, antioxidant, Hp, hemolysis assay, silver nanoparticles

## Abstract

Bioactive molecule-based synthesis of silver nanoparticles (AgNPs) offers an eco-friendly approach with high therapeutic potential; however, research in this area remains limited. This study introduces hot melt extrusion (HME) technology to enhance the extraction efficiency of bioactive compounds, including astaxanthin, from the microalgae *Haematococcus pluvialis* (Hp). AgNPs were synthesized using HME-processed Hp (H-Hp/AgNPs), confirmed by a color change and UV–vis absorption spectrum. The resulting H-Hp/AgNPs exhibited an average size of 129.7 ± 10.4 nm, a polydispersity index of 0.2 ± 0.3, and a zeta potential of −31.54 ± 0.2 mV, indicating high stability. The synthesized AgNPs demonstrated antibacterial activity by inhibiting the growth and biofilm formation of antibiotic-resistant bacteria. Cell viability assays revealed that normal cells maintained over 100% viability at most concentrations of H-Hp/AgNPs, while cancer cells exhibited significant cytotoxicity (34.1 ± 3.1%) at 250 μg/mL. Furthermore, H-Hp/AgNPs induced apoptosis in MDA-MB 231 cells, as evidenced by mitochondrial membrane potential loss, nuclear condensation, and apoptosis, confirmed through AO/EB, Rh123, and PI staining. Additionally, H-Hp/AgNPs showed no hemolytic activity at concentrations below 250 μg/mL, ensuring safety. In conclusion, this study highlights the potential of biosynthesized H-Hp/AgNPs as promising candidates with antioxidant, antibacterial, biocompatibility, and anticancer properties.

## 1. Introduction

Silver nanoparticles (AgNPs) have been widely studied due to their excellent physicochemical and biological properties and exhibit various therapeutic effects such as anticancer, antidiabetic, antioxidant, antibacterial, and anti-inflammatory activities [1, 2]. It has particularly strong antibacterial properties and is considered a potential alternative to avoid antibiotic resistance [3]. AgNPs can be synthesized using chemical, biological, and physical methods [4]. Among these, biological synthesis utilizes natural resources such as bacteria, plants, yeast, fungi, and algae and is preferred due to its cost-effectiveness and environmentally friendly nature [5]. Several studies have been conducted on the biosynthesis of nanoparticles using seaweed extracts. Microalgae are one of the most promising bioresources for the production of nanomaterials and are attracting the attention of researchers as they have the potential to develop new anticancer drugs [6, 7]. Synthesis of AgNPs using microalgae is considered advantageous due to its rapid and cost-effective nature and its ability to produce nanoparticles with enhanced biocompatibility and therapeutic potential [8].

Natural biomaterials present in seaweed aqueous extracts, such as flavonoids, terpenoids, proteins, carbohydrates, and lipids [9–11], act as capping and reducing agents to reduce silver ions for AgNPs' production [12]. Among them, flavonoids play an important role in the reduction process through tautomerization, where the enol form is converted to the keto form, releasing reactive hydrogen atoms and donating electrons to silver ions to promote the formation of AgNPs [13]. In addition, enzymes, biomolecules, and functional groups such as hydroxyl, carboxyl, and amino contribute to the reduction and stabilization mechanisms, thereby enhancing the synthesis process and preventing nanoparticle aggregation [14]. Although the use of microalgae in nanoparticle synthesis is very limited, some studies have reported promising applications of microalgae for metal nanoparticle synthesis [15]. Therefore, this study aims to develop a sustainable and efficient approach for the biosynthesis of AgNPs using bioactive molecules extracted from *Haematococcus pluvialis* (Hp). By incorporating hot melt extrusion (HME) technology, we seek to enhance the extraction efficiency of key bioactive compounds, including astaxanthin, to facilitate AgNPs' synthesis. The study further evaluates the therapeutic potential of these nanoparticles, particularly in biomedical applications.

Hp is a unicellular biflagellate green microalgae distributed worldwide in many aquatic habitats [16]. It is known to produce bioactive compounds such as proteins, carotenoids, and astaxanthin [17], astaxanthin comprising up to 2%-3% of its dry weight, surpassing other microbial sources [18]. Astaxanthin, a potent antioxidant, exhibits up to tenfold higher activity than other carotenoids and has been widely explored for its pharmacological applications, including its role in oxidative stress regulation, immune modulation, and potential anticancer activity [19, 20]. Hp cells stop dividing and produce larger and thicker cell walls when exposed to intense solar radiation, nutrient deprivation, or other stressors [21]. These conditions cause the Hp cell wall to become increasingly thick, making it highly resistant to chemical and physical disruption efforts [22]. This structural resistance, however, limits the bioavailability and efficient extraction of intracellular bioactive molecules such as astaxanthin [23]. From this perspective, strategies are needed to increase the extraction efficiency of valuable intracellular products, such as proteins, carotenoids, and especially astaxanthin, from Hp cells.

HME is a continuous process in which polymeric materials are pumped through a rotating screw at elevated temperatures through a die into a uniformly shaped product [24]. It is used in the pharmaceutical industry to produce drug delivery systems and dosage forms with properties such as improved solubility of poorly water-soluble compounds [25], controlled release [26], sustained and targeted drug delivery [27], and uniformity of extrudates [28]. HME technology has recently been introduced into natural product processing and is used to improve the water solubility of hydrophobic compounds and the solubility and bioavailability of poorly soluble drugs by using appropriate excipients [29, 30]. Furthermore, previous studies have reported the anticancer effects of Hp extracts [31], suggesting that AgNPs synthesized from these extracts could be ideal mediators to target cancer cells.

However, the exact role of Hp-derived biomolecules in nanoparticle formation and their impact on biological activities are still largely unexplored. To the best of our knowledge, this is the first study to explore the biosynthesis of AgNPs using Hp extracts treated with HME and their various biological activities. UV–vis spectroscopy, dynamic light scattering (DLS), Fourier-transform infrared spectroscopy (FT-IR), X-ray diffraction (XRD), transmission electron microscopy (TEM), and energy-dispersive X-ray spectroscopy (EDS) were used to comprehensively characterize these nanoparticles. The main objectives of this study were to evaluate the antioxidant capacity of H-Hp/AgNPs, to evaluate their antimicrobial activity against gram-positive, gram-negative, and antibiotic-resistant bacteria, to confirm their biocompatibility through red blood cell hemolysis assay, and to determine their anticancer potential against human breast cancer cells (MDA-MB-231) while ensuring minimal cytotoxicity against normal cells (HaCaT). This study presents a sustainable and efficient method for producing biologically active AgNPs with enhanced therapeutic potential by integrating HME technology with nanoparticle biosynthesis.

## 2. Materials and Methods

### 2.1. Materials

Potassium acetate (CH_3_CO_2_K, CAS number 7447-40-7, ≥ 99%) and gallic acid (C_7_H_6_O_5_, CAS number 149-91-7, ≥ 97.5%) were obtained from Daejung Chemical (Siheung, Republic of Korea). Folin–Ciocalteu's phenol reagent (CAS number 12111-13-6) and quercetin (C_15_H_10_O_7_, CAS number 117-39-5, ≥ 95%) were obtained from Sigma-Aldrich (St. Louis, MO, USA). Mueller–Hinton broth (MHB) and Mueller–Hinton agar (MHA) were purchased from MB cell (Gifco, USA). Dulbecco's modification of Eagle's medium (DMEM), phosphate-buffered saline (PBS), and fetal bovine serum (FBS) were purchased from Thermo Fisher Scientific (Massachusetts, USA). Penicillin–streptomycin was purchased from Cytiva (Incheon, Republic of Korea). Dimethyl sulfoxide (DMSO, > 99.7%) for cell culture was purchased from Gen DEPOT (Altair, TX, USA). Sheep blood defibrinated was obtained from KisanBio (Seoul, Korea). WST assay kits were purchased from CelloMax (Seoul, Republic of Korea). Acridine orange (AO) (C_17_H_19_N_3_ HCl, CAS number 65-61-2), ethidium bromide (EB) (C_21_H_20_BrN_3_, CAS number 1239-45-8), and propidium iodide (PI) (C_27_H_34_I_2_N_4_, CAS number 25535-16-4) were purchased from Sigma-Aldrich (St. Louis, MO, USA). Rhodamine 123 (Rh123) (C_21_H_17_C_l_N_2_O_3_, CAS number 62669-70-9) and Annexin V-FITC/PI apoptosis detection kit were obtained from Invitrogen (Vienna, Austria).

### 2.2. Preparation of Plant Extract

Hp powder and each excipient were mixed, and the HME process (STS-25H twin-screw extruder, EM Korea Co., Ltd., Pyeongtaek, Korea) was performed. The excipients hydroxypropyl-beta-cyclodextrin (HPCD), lecithin, ascorbyl palmitate, hydroxypropyl methylcellulose (HPMC), vitamin C, and vitamin E were mixed in a specific ratio and used (Table 1). The treatment temperature was fixed at 60°C. The HME process conditions were 15 bar pressure and 180 rpm screw speed. After completion of the HME process, the extrudate was dried in an oven at 60°C for 48 h, pulverized, and powdered. Then, 1 g of non-HME-Hp (N-Hp) and H-Hp powder were added to 50 mL of distilled water (DW), followed by ultrasonic extraction at 40°C for 1 h. The extract was filtered through the Whatman 6 filter paper at room temperature. The aqueous extract was stored at 4°C for later use.

### 2.3. Synthesis of AgNPs

Silver nitrate (AgNO_3_) solutions of different concentrations (1, 3, 5, 10, and 15 mM) were mixed with N-Hp and H-Hp. In addition, H-Hp and AgNO_3_ solutions were mixed at ratios of 1:9, 3:7, 5:5, 7:3, and 9:1 to find the optimal synthesis conditions. The mixture was incubated at 60°C and stored for 24 h. The mixture was centrifuged at 13,000 rpm for 20 min. DW was added repeatedly, and a washing process was performed three times to remove silver ions and extract residues from the pellet.

### 2.4. Characterization

Analysis of AgNPs' synthesis was involved using a UV–vis spectrometer (Optizen Pop, Daejeon, Korea) at 300–700 nm using a cuvette cell containing 1 mL of the sample. Particle size, polydispersity index (PDI), and zeta potential were measured with a Mastersizer 2000 (Malvern Instruments, Malvern, UK). Morphological details were observed by loading AgNP samples on copper grids and employing TEM with EDS capabilities (JEM-2100F, JEOL, Tokyo, Japan). The crystalline structure of AgNPs was analyzed using XRD patterns over 2*θ* angles ranging from 5° to 80°, employing an X-ray diffractometer (MPD, PAN Analytical, The Netherlands). The functional characteristics of H-Hp/AgNPs were analyzed in FT-IR (PerkinElmer Paragon 500 USA).

### 2.5. Phytochemical and Antioxidant Analysis

#### 2.5.1. Total Phenolic Content (TPC)

The TPC of H-Hp and H-Hp/AgNPs was quantified using the Folin–Ciocalteu colorimetric method, based on the oxidation–reduction principle, with slight modifications as per the previous report [32]. For the nanoparticles, samples (1 mg/mL) were initially immersed in 99% ethanol for 2 h and then centrifuged at 1000 rpm, and the supernatant was used for analysis. Briefly, 0.2 mL of the sample (1 mg/mL) was mixed with 0.6 mL of DW in a test tube. Then, 1 mL of Folin–Ciocalteu reagent and 0.8 mL of 7.5% sodium carbonate solution were added to the mixture. The solution was incubated at room temperature for 45 min, after which the absorbance was measured at a wavelength of 760 nm. Gallic acid solution was used as a reference standard, and the concentrations were 31.3, 62.5, 125, 250, and 500 μg/mL.

#### 2.5.2. Total Flavonoid Content (TFC)

The TFC of H-Hp and H-Hp/AgNPs (sample preparation as mentioned in the above section) was measured using a modified aluminum chloride colorimetric method, as described in an earlier study [33]. Briefly, 400 μL of the sample (1 mg/mL) was combined with 20 μL of 10% aluminum chloride and 20 μL of 1 M potassium acetate in a test tube. Subsequently, 1 mL of DW was added to the mixture, which was then allowed to react for 30 min. The absorbance of the resulting solution was measured at a wavelength of 415 nm. For calibration, a series of quercetin standard solutions at concentrations of 31.3, 62.5, 125, 250, and 500 μg/mL were utilized.

#### 2.5.3. 2,2-Diphenyl-1-Picrylhydrazyl (DPPH) Assay

The scavenging activity of DPPH radicals was assessed using a slightly modified method, as described in the previous report [34]. To measure the scavenging activity, the various concentration extracts and the H-Hp/AgNPs (200, 400, 600, 800, and 1000 μg/mL) were mixed with 0.4 mM DPPH solution in a 1:1 ratio. The mixture was incubated in the dark at room temperature for 20 min. After incubation, the absorbance was measured at a wavelength of 517 nm. Ascorbic acid was used as the standard, with concentrations of 20, 40, 60, 80, and 100 μg/mL for comparison.

#### 2.5.4. 2,2′-Azino-Bis (3-Ethylbenzothiazoline-6-Sulfonic Acid) (ABTS) Assay

The scavenging activity of ABTS radicals was measured following published methods, as detailed in a previous report [35]. A solution of potassium persulfate and ABTS^+^ was prepared, with final concentrations of 2.45 mM and 7 mM, respectively, and mixed in a 1:1 ratio. The mixture was allowed to react in the dark for 16 h. Following this, the solution was diluted with 0.1 M PBS at pH 7.4 until an absorbance of 0.9 at 734 nm was achieved. For the assay, a mixture of 30 μL of the extract and the H-Hp/AgNPs (200, 400, 600, 800, and 1000 μg/mL) and 150 μL of the ABTS solution was prepared and incubated in the dark for 25 min. The absorbance was then measured at 734 nm. Ascorbic acid was used as the standard according to the above section.

### 2.6. Antibacterial Assays

#### 2.6.1. Well Diffusion Assay

*Bacillus cereus* (*B. cereus*) (American Type Culture Collection (ATCC) 14579), *Listeria monocytogenes* (*L. monocytogenes*) (ATCC 15313), *Staphylococcus aureus* (*S. aureus*) (ATCC 19095), *Salmonella enterica* (*S. enterica*) (ATCC 14028), *Escherichia coli* (*E. coli*) (ATCC 43888), and methicillin-resistant *Staphylococcus aureus* (MRSA) (ATCC 43300) for bacterial cultures were bought from ATCC. To evaluate the antibacterial activity of AgNPs, an agar well diffusion method was used, with some modifications to the previous report [36]. Briefly, 100 μL of each bacteria was inoculated into 10 mL of sterilized MHB medium and cultured at 37°C for 24 h. The bacterial cultures were plated on MHA on a petri dish. A metal cork borer was then used to create a well in the dish. H-Hp/AgNPs were diluted to 250, 500, and 1000 μg/mL with PBS, and 50 μL of each concentration sample was added to each well. It was compared with the tetracycline hydrochloride (TCH) for positive control. Additionally, all petri dishes were incubated at 37°C for 24 h. The zones of inhibition (ZOI) of each well were then calculated and compared.

#### 2.6.2. Minimum Inhibitory Concentration (MIC) and Minimum Bactericidal Concentration (MBC)

The MIC and MBC were assessed using a slightly modified method, as described in the earlier study [37]. Bacterial suspensions were grown in MHB and incubated at 37°C for 24 h. Various concentrations (15.6, 31.3, 62.6, 125, 250, 500, and 1000 μg/mL) of H-Hp/AgNPs were prepared by serial dilution in MHB medium. The bacterial suspension was diluted in MHB to an absorbance of 0.1 at 600 nm. Then, 100 μL of AgNP suspension and 100 μL of bacterial inoculum were added to each well. Bacterial growth was observed at an optical density (OD) of 600 nm using a UV–vis spectrophotometer for various times (0, 2, 4, 6, 8, 12, and 24 h) up to 24 h compared to control cells. The MBC value of the biosynthesized nanoparticles was determined through solid medium MHA cultivation. And, 50 μL of samples cultured 1:1 with bacteria for 24 h were aliquoted into MHA and incubated at 37°C for 24 h, and colony-forming unit (CFU) values were confirmed.

#### 2.6.3. Antibiofilm Assay

The antibiofilm assay was assessed using a slightly modified method, as described in the previous report [38]. The antibiofilm properties of H-Hp/AgNPs were confirmed against *S. aureus*, *E. coli*, *L. monocytogenes*, *S. enterica*, *B. cereus*, and MRSA. For biofilm analysis, 100 μL of bacterial pathogens cultured in MHB were poured into a 96-well plate, and 100 μL of various concentrations of H-Hp/AgNPs were added and cultured at 37°C for 24 h. PBS was used as a negative control. The medium containing bacteria was carefully removed from the well and the attached bacterial cells were rinsed with PBS until no particles were visible. Then, 200 μL of 0.1% crystal violet was added to the attached cells and fixed in the well for 30 min. In addition, each well was carefully washed with PBS to remove unbound crystal violet. The plates were then inverted and air-dried for 60 min at room temperature. To quantify biofilms, 200 μL of ethanol was added to dried wells to dissolve the crystal violet associated with adherent cells and evaluated at 600 nm.

### 2.7. Anticancer

#### 2.7.1. Cell Viability (%)

The cell viability properties of H-Hp/AgNPs were assessed on normal cells (HaCaT) and breast cancer cells (MDA-MB 231). HaCaT and MDA-MB 231 cells were cultured in DMEM (10% FBS, 1% P/S) medium and maintained in a 5% CO_2_ incubator at 37°C. For the experiment, HaCaT and MDA-MB 231 cells (1 × 10^4^/well) of 100 μL were added to a 96-well plate and stored in a 5% CO_2_ incubator for 24 h. Then, 10 μL of various concentrations (19.5, 31.3, 62.5, 125, 250, 500, and 1000 μg/mL) of H-Hp/AgNPs were added to a 96-well plate and the culture continued for 24 h. Then, 10 μL of WST solution was added to each well and the cells were incubated for 1 h, the plate was measured at 450 nm with a UV–vis spectrophotometer, and cell viability (%) was calculated.

(1) $$\begin{array}{c} \text{Cell viability}\left(\%\right)=\frac{\text{OD value of samples}}{\text{OD value of controls}}\times 100. \end{array}$$

#### 2.7.2. Cell Staining

MDA-MB 231 cells were seeded in a 24-well plate at a density of 2 × 10^5^ cells/well in a 5% CO_2_ incubator at 37°C and cultured for 24 h. A control (PBS) and H-Hp/AgNPs were treated in cultured cells for 24 h. The cells were washed with PBS, and 10 μL of AO/EB, Rh123, and PI were added to each well and incubated for 1, 15, and 15 min, respectively. Afterward, it was washed once more with PBS and examined under a fluorescence microscope at 20x magnification.

#### 2.7.3. Apoptosis Assay

Apoptosis assays for MDA-MB cells were performed according to the protocol of the manufacturer. MDA-MB 231 cells (1 × 10^5^ cells/well) were cultured in a 6-well plate at 37°C for 24 h. Then, with 250 μg/mL of H-Hp AgNPs, it was treated for 24 h. Cells were washed with PBS and resuspended in 195 μL of 1x binding buffer. After centrifugation at 1300 rpm for 5 min, 5 μL of Annexin V-FITC was added and incubated for 10 min at room temperature. Then, 10 μL of PI was added, and the analysis was performed using a flow cytometer (Becton Dickinson, USA).

### 2.8. Hemolysis Assay

The blood compatibility of H-Hp/AgNPs was assessed in a hemolysis assay following the previous method [39]. Specifically, 1 mL of defibrinated sheep blood was mixed with 9 mL of PBS. Then, it was centrifuged at 2000 rpm for 10 min, washed three times with PBS, and carefully dispersed in 10 mL of PBS. Then, various concentrations of H-Hp/AgNPs (200 μL) were incubated with red blood cells (200 μL) at 37°C for 1 h, and then the reaction solution was centrifuged at 2000 rpm for 10 min to precipitate the red blood cells. The absorbance of the supernatant was measured at 540 nm using a UV–vis spectrophotometer. And, 1% Triton X-100 was used as a positive control, and PBS was used as a negative control. The hemolysis rate of H-Hp/AgNPs was then calculated compared to the control group.

### 2.9. Statistical Analysis

The data were analyzed using two-way analysis of variance (ANOVA) for comparison. All experimental analyses were performed in triplicate. The Duncan multiple range test (DMRT) was employed for statistical analysis of the results (*p* < 0.05).

## 3. Results and Discussion

### 3.1. Characterization

#### 3.1.1. Color Change and UV–vis Spectrometer

**Figure 1:**
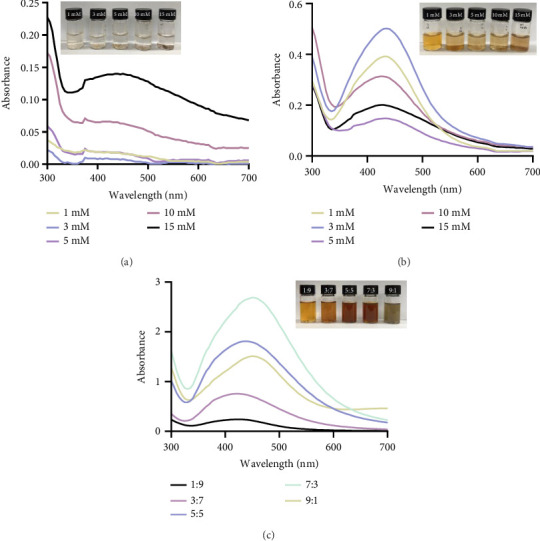
UV–vis spectrum of N-Hp for different concentrations of AgNO_3_ at 24 h (a); for different concentrations at 24 h (b); for different ratios of H-Hp and AgNO_3_ at 24 h (c).

Initial screening for AgNPs during synthesis was performed using UV–vis spectral analysis. UV–vis analysis is used to obtain information about AgNPs such as stability in size and shape stability in aqueous suspensions [40]. The reason that size and shape information can be obtained is that the wavelength range of the peak can vary depending on the particle size and shape of the AgNPs [41].  Figure 1 shows the absorption spectra of N-Hp and H-Hp mixed with AgNO_3_ solution. N-Hp was mixed with AgNO_3_ solutions of various concentrations (Figure 1(a)). The presence of AgNPs can be correlated with the UV–vis spectrum [42]. The absence of clear peaks and no color change indicates that AgNPs were not synthesized, indicating that the reduction of AgNO_3_ did not occur under the reaction conditions used [43]. On the other hand, the color change and the presence of a peak at 420 nm indicate the surface plasmon resonance (SPR) phenomenon induced by AgNPs [44]. The appearance of distinct peaks in Figures 1(b) and 1(c) shows the SPR characteristics of AgNPs, indicating the formation of AgNPs. It was synthesized with AgNO_3_ solutions of various concentrations, and the highest peak for 3 mM AgNO_3_ solution appeared 24 h after synthesis (Figure 1(b)). It was confirmed that absorbance did not increase with increasing AgNO_3_ concentration. The nondependence on the increasing concentration of H-Hp/AgNPs may be due to the particle size formation during the synthesis of AgNPs from Hp extract using AgNO_3_ [45]. This may be due to the increase in the density of the nanoparticles with increasing concentration. In addition, various ratios of H-Hp and 3 mM AgNO_3_ were synthesized (Figure 1(c)), and a shift in the SPR peak was observed, indicating changes in the size and aggregation state of the nanoparticles with varying ratios [46]. At a ratio of 1:9, relatively small-sized AgNPs were formed, resulting in a peak at a lower wavelength. Conversely, at a ratio of 9:1, the particles appeared to be larger, shifting the peak to a higher wavelength. This observation aligns with a previous study that confirmed a redshift in wavelength as particle size increased [47]. The highest peak was observed at a 7:3 ratio after 24 h of synthesis. Thus, this concentration and ratio were selected for the further synthesis of AgNPs in this study.

#### 3.1.2. FT-IR and XRD

**Figure 2:**
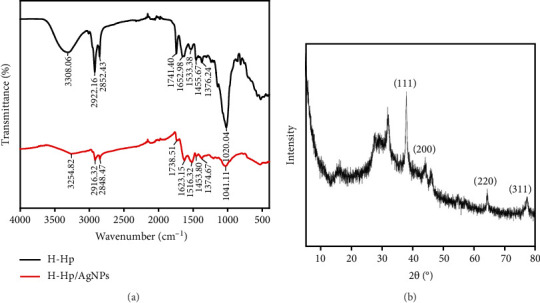
FT-IR spectrum of H-Hp and H-Hp/AgNPs (a); XRD spectrum of H-Hp/AgNPs (b).

FT-IR spectroscopy analysis was conducted to identify the functional groups present in H-Hp and H-Hp/AgNPs, with spectra recorded between 4000 cm^−1^ and 400 cm^−1^ (Figure 2(a)). The FT-IR spectrum of H-Hp revealed key peaks at 3308 cm^−1^, 2922 cm^−1^, 2852 cm^−1^, 1741 cm^−1^, 1653 cm^−1^, 1533 cm^−1^, 1456 cm^−1^, 1376 cm^−1^, and 1020 cm^−1^. The peak at 3308 cm^−1^ corresponds to O–H stretching vibrations, indicating the presence of phenolic and hydroxyl groups. It has been reported that phenolic compounds in aqueous extracts can reduce AgNO_3_, acting as a capping and stabilizing agent [48]. The peaks at 2922 cm^−1^ and 2852 cm^−1^ were assigned to C–H stretching vibrations of alkanes, while the peak at 1741 cm^−1^ corresponds to C=O stretching of carbonyl groups, which may facilitate the reduction of silver (Ag^+^) ions [49]. The 1653 cm^−1^ peak suggests the presence of C=O groups from aldehydes, ketones, carboxylic acids, and esters. The peak at 1533 cm^−1^ suggests the presence of amide II vibrations, likely originating from proteins that can interact with AgNPs, whereas the peak at 1456 cm^−1^ corresponds to C–N stretching, and the peak at 1020 cm^−1^ is attributed to the C–O–C stretching of ether groups.

The FT-IR spectrum of H-Hp/AgNPs displayed major peaks at 3255 cm^−1^, 2916 cm^−1^, 2858 cm^−1^, 1738.51 cm^−1^, 1623 cm^−1^, 1516 cm^−1^, 1454 cm^−1^, 1375 cm^−1^, and 1041 cm^−1^, with notable shifts and intensity changes compared to H-Hp, indicating interactions between functional groups and AgNPs. The broadening and shifting of the O–H stretching peak from 3308 cm^−1^ to 3255 cm^−1^ suggest that the hydroxyl group actively participated in the reduction of silver ions and acted as a capping agent to prevent nanoparticle aggregation [50]. The shift of the C=O stretching peak from 1741.40 cm^−1^ to 1738.51 cm^−1^ and from 1653 cm^−1^ to 1623 cm^−1^ and the decrease in intensity suggest that the carbonyl group interacted and bound with the AgNPs' surface, which further supports the role of AgNPs in the surface stabilization process of nanoparticles [51, 52]. The shift of the amide II band from 1533 cm^−1^ to 1516 cm^−1^ suggests that there may be interactions between AgNPs and protein residues in the extract, which may enhance the nanoparticle dispersion and colloidal stability. The shift of the ether-related peak from 1020 cm^−1^ to 1041 cm^−1^ and the significant decrease in intensity suggest that the C–O–C ether bond strongly interacted with the AgNP surface, contributing to the change in surface properties. In particular, the decrease in the peak suggests that the ether group plays a role in enhancing the stability and dispersibility of nanoparticles by binding through interactions with the AgNP surface, which is consistent with previous reports suggesting that surface functional groups of nanoparticles contribute to stabilization [53]. These results indicate that the H-Hp/AgNPs were successfully synthesized, and the biologically active compounds of H-Hp not only promoted the AgNPs' formation but also ensured long-term colloidal stability through electrostatic interactions and functional group modification.

The crystalline nature of H-Hp/AgNPs was confirmed through XRD pattern analysis (Figure 2(b)). The XRD analysis revealed the presence of four prominent diffraction peaks at specific 2*θ* values: 38° (111), 44° (200), 64° (220), and 77° (311). These sharp peaks may be caused by the capping agent stabilizing the nanoparticles [54]. This peak has a cubic structure consistent with the face-centered cubic information of file number 00-004-0783 provided by the Joint Committee on Powder Diffraction Standards (JCPDS) and was confirmed to be due to the crystallographic plane of H-Hp/AgNPs [55].

#### 3.1.3. TEM, EDS, DLS, and Zeta Potential

**Figure 3:**
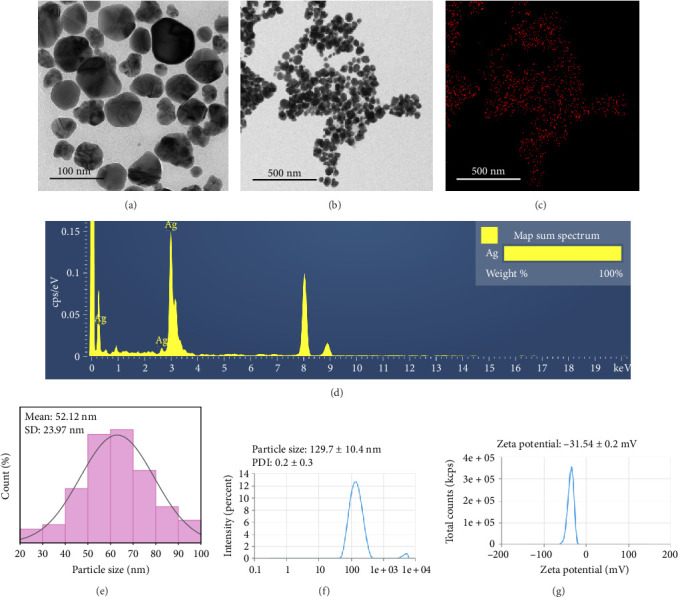
TEM image of H-Hp/AgNPs. H-Hp/AgNPs with different magnification (a, b); EDS mapping of Ag (c); EDS spectrum of H-Hp/AgNPs (d); histogram of particle size distribution of H-Hp/AgNPs analyzed by ImageJ software (e), particle size (nm), and PDI of H-Hp/AgNPs (f); and zeta potential (mV) of H-Hp/AgNPs (g).

TEM images provide information about the size, morphology, and shape of the biosynthesized H-Hp/AgNPs [56]. TEM analysis was performed using scale bars of 100 nm and 500 nm, revealing that the H-Hp/AgNPs exhibited irregular shapes (Figures 3(a) and 3(b)). The shape, size, and other characteristics may have been influenced by the physicochemical parameters of the composition [57]. In Figure 3(c), H-Hp/AgNPs were synthesized using H-Hp extract as a reducing and stabilizing agent, and the presence of silver was confirmed. The strong and distinct peak around 3 keV for silver in the EDS spectrum indicates the successful formation of AgNPs (Figure 3(d)). To determine the size distribution of H-Hp/AgNPs, approximately 100 particles were analyzed and represented in a histogram. Figure 3(e) presents the particle size distribution histogram of H-Hp/AgNPs, indicating an average particle size of 52.12 ± 23.97 nm.

The particle size, PDI, and zeta potential of H-Hp/AgNPs were measured. As a result of performing DLS to measure the size distribution of AgNPs, the particle size of H-Hp/AgNPs was measured to be 129.7 ± 10.4 nm (Figure 3(f)). DLS, an indirect ensemble technique based on the luminosity signal of particles, is influenced by particle structure and radius-dependent scattering and is biased toward larger sizes [58, 59]. Therefore, the diameter obtained by DLS is generally larger than the diameter measured by TEM [60]. A previous study reported that AgNPs synthesized from *Tabebuia roseo-alba* had a particle size of 81.04 nm as measured by DLS analysis, whereas the particle diameter determined by TEM was 8.7 ± 0.21 nm, revealing a discrepancy of approximately tenfold [61]. For PDI values > 0.7, the sample is considered to be polydisperse [62]. AgNPs were measured to have a PDI of 0.2 ± 0.3, which makes them relatively monodisperse. The stability of AgNPs is mainly determined by the surface charge through the measurement of zeta potential [63]. Particles with zeta potential values below −30 mV are classified as stable by determining the repulsion between particles [64]. The zeta potential value of H-Hp/AgNPs was −31.54 ± 0.2 mV, confirming that the particles were in a stable state (Figure 3(g)).

### 3.2. Phytochemical and Antioxidant Analysis

#### 3.2.1. TPC and TFC

**Figure 4:**
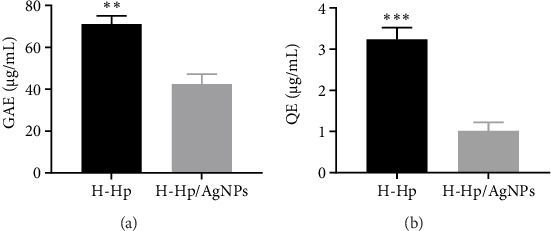
TPC (a); and TFC (b) of H-Hp and H-Hp/AgNPs. The data are presented as the mean ± standard deviation (SD) (*n* = 3), and difference evaluations are shown as ^∗∗^*p* < 0.01 and ^∗∗∗^*p* < 0.001.

Figure 4 shows the TPC and TFC results of the H-Hp extract and H-Hp/AgNPs. The TPC was 71.03 ± 4 mg/g for H-Hp extract and 42.47 ± 4.78 mg/g for H-Hp/AgNPs. The TFC was 3.24 ± 0.29 mg/g for H-Hp extract and 1.01 ± 0.21 mg/g for H-Hp/AgNPs. Polyphenolic components can ensure broad bioactivity by interacting with AgNPs as reducing and capping agents [65]. Algae are attracting attention as a sustainable and rich source of bioactive compounds such as phenolic compounds, fatty acids, amino acids, and carotenoids [66]. Microalgae can be an alternative source of natural antioxidants because they are much more diverse than other sources such as plants [67]. The biologically active compounds in the H-Hp extract are incorporated onto the surface of AgNPs during the reduction of silver ions (Ag^+^) to metallic silver (Ag^0^) nanoparticles. Due to the large surface area of the nanoparticles, they can absorb a greater quantity of active compounds. However, the TPC and TFC values in H-Hp/AgNPs were lower compared to those in H-Hp extracts. This suggests that polyphenolic compounds may be involved in the reduction and capping process of H-Hp/AgNPs. Similar results have been reported in other studies as changes in TPC and TFC values [68, 69].

#### 3.2.2. ABTS and DPPH

**Figure 5:**
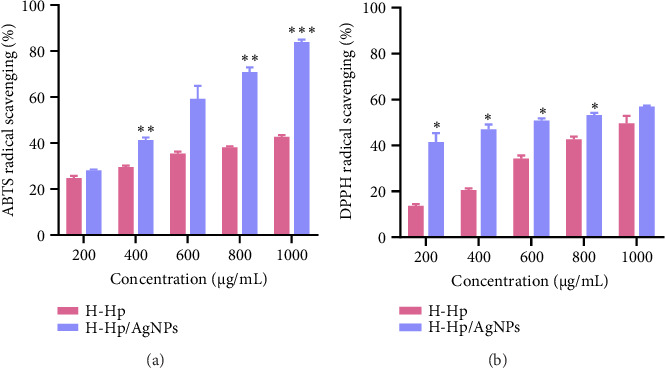
Antioxidant activity of H-Hp extract and H-Hp/AgNPs. ABTS (a); and DPPH (b). The data are presented as the mean ± SD (*n* = 3), and difference evaluations are shown as ^∗^*p* < 0.05, ^∗∗^*p* < 0.01, and ^∗∗∗^*p* < 0.001.

The ABTS and DPPH radical scavenging rates (%) of H-Hp extract and H-Hp/AgNPs are shown in Figure 5. The IC_50_ values for ABTS radical scavenging activity were 53.36 ± 2.96 μg/mL for ascorbic acid (Figure S1), 1314.16 ± 38.36 μg/mL for the H-Hp extract, and 504.98 ± 21.04 μg/mL for H-Hp/AgNPs (*p* < 0.001). This indicates that H-Hp/AgNPs exhibited significantly improved radical scavenging ability compared to the H-Hp extract, although they were less effective than ascorbic acid. Notably, the ABTS radical scavenging rate (%) increased in a concentration-dependent manner, with H-Hp/AgNPs consistently demonstrating higher activity than the H-Hp extract (Figure 5(a)). Similarly, the IC_50_ values for DPPH radical scavenging activity were 23.67 ± 3.88 μg/mL for ascorbic acid (Figure S1), 982.09 ± 51.63 μg/mL for the H-Hp extract, and 592.73 ± 63.71 μg/mL for H-Hp/AgNPs. A significant reduction in IC_50_ was observed in H-Hp/AgNPs compared to the H-Hp extract (*p* < 0.01), indicating enhanced radical scavenging potential. The DPPH radical scavenging rate (%) also increased in a concentration-dependent manner, with H-Hp/AgNPs exhibiting significantly higher activity than the H-Hp extract at all tested concentrations (Figure 5(b)). Phenolic and flavonoid compounds are important phytochemicals with antioxidant abilities to deactivate free radicals. H-Hp/AgNPs biosynthesized using H-Hp extract exhibited scavenging activity due to the phenolic compounds capped on the surface. The interaction between the antioxidant compounds in AgNPs and the H-Hp extract enhanced antioxidant activity and promoted the free radical scavenging reaction. Antioxidant compounds, such as phenols and flavonoids, adsorbed on the surface of AgNPs, not only stabilized free radicals through their electron-donating ability but also enabled AgNPs to act as singlet oxygen scavengers and hydrogen donors [70]. These findings are consistent with previously reported DPPH and ABTS free radical scavenging activities of AgNPs, which further support our results [71, 72].

### 3.3. Antibacterial Efficacy

#### 3.3.1. Well Diffusion Assay, MIC, and MBC

**Figure 6:**
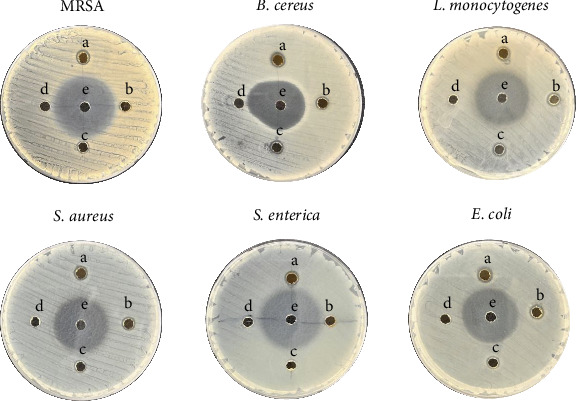
Antibacterial activity of H-Hp/AgNPs on different bacterial pathogens determined in well diffusion assay (H-Hp/AgNPs a-1000 μg/mL; b-500 μg/mL; c-250 μg/mL; H-Hp extract d-1000 μg/mL; and TCH e-1000 μg/mL).

The antibacterial activity of H-Hp/AgNPs was evaluated against gram-positive bacteria (*L. monocytogenes, S. aureus, B. cereus*), gram-negative bacteria (*E. coli, S. enterica*), and antibiotic-resistant bacteria (MRSA). The ZOI for various concentrations of H-Hp, H-Hp/AgNPs, and positive control is shown in Figure 6 and Table 2. The results showed that no ZOI was observed at 1000 μg/mL of H-Hp extract treatment, while H-Hp/AgNPs showed concentration-dependent antibacterial activity, confirming that AgNPs were the main cause of the antibacterial effect. H-Hp/AgNPs (1000 μg/mL) formed ZOI of MRSA (8.2 ± 0.3 mm), *B. cereus* (10.2 ± 0.8 mm), *L. monocytogenes* (9.2 ± 0.8 mm), *S. aureus* (9.3 ± 0.6 mm), *E. coli* (9.7 ± 0.6 mm), and *S. enterica* (9.8 ± 0.7 mm) (Table 2). However, ZOI was not observed at all concentrations. In the case of *S. enterica*, ZOI was not observed at 250 and 500 μg/mL, indicating that the antibacterial effect on specific strains could be confirmed by differences in sensitivity depending on the concentration and characteristics of the bacteria. Meanwhile, H-Hp/AgNPs showed the strongest antibacterial effect against *B. cereus*, recording the largest ZOI in the gram-positive bacteria *B. cereus*.

Although the ZOI of H-Hp/AgNPs was relatively small, it is noteworthy that antibacterial activity was observed even at low concentrations (250 and 500 μg/mL). The irregular shape and surface properties of AgNPs affect the antibacterial activity and mechanism [73]. In general, spherical nanoparticles are expected to have increased antibacterial activity by contacting bacteria with a uniform distribution. However, the more irregular the surface, the greater the contact area with bacteria, which can actively interact with the cell membrane and promote the release of silver (Ag^+^) ions. In previous studies, it was reported that the antibacterial activity of various shapes of AgNPs was compared, and even if the shape of the particles was irregular, they could have different effective surface areas for the active aspect and show different antibacterial effects [74, 75]. Therefore, it is thought that H-Hp/AgNPs maintained antibacterial activity even at low concentrations because the contact area with bacteria increased.

These variations in antibacterial activity suggest that not only the shape of AgNPs but also bacterial cell wall composition plays a crucial role in determining susceptibility, as differences in membrane structure can influence how nanoparticles interact with and penetrate bacterial cells. Many studies have shown that nanoparticles have greater activity against gram-positive bacteria than against gram-negative bacteria [76]. While the outer membrane of gram-negative bacteria (LPS, lipoproteins, and phospholipids) physically restricts the penetration of nanoparticles, gram-positive bacteria have a porous peptidoglycan layer and a negatively charged cell wall, which facilitate electrostatic interactions with AgNPs and facilitate their penetration [77].

**Figure 7:**
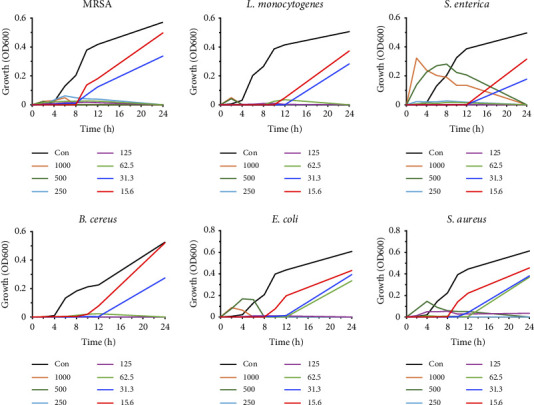
Growth kinetics of different bacterial pathogens alone and along with different concentrations of H-Hp/AgNPs.

Bacterial growth using different concentrations of H-Hp/AgNPs was measured at various time intervals up to 24 h (Figure 7). The results confirmed that H-Hp/AgNPs inhibited bacterial growth at 62.5 μg/mL within 24 h for MRSA, *L. monocytogenes*, *S. enterica*, and *B. cereus*. In addition, bacterial growth did not occur below 62.5 μg/mL for *E. coli* and 125 μg/mL for *S. aureus* within 24 h. Therefore, the MIC values for MRSA, *L. monocytogenes*, *S. enterica*, *B. cereus*, *E. coli*, and *S. aureus* were 62.5, 62.5, 62.5, 62.5, 125, and 250 μg/mL, respectively. Additionally, the MBC values of 125, 62.5, 62.5, 62.5, 250, and 250 μg/mL were observed (Table 3). On the other hand, H-Hp extract had MIC of 50, 40, 40, 40, 50, and 50 mg/mL against MRSA, *L. monocytogenes*, *S. enterica*, *B. cereus*, *E. coli*, and *S. aureus*, and MBC was not shown at the given concentrations. The antibacterial properties of AgNPs against bacterial strains have been the subject of several studies, but their exact mode of action has not been fully described. Studies have shown that the strong antibacterial properties of AgNPs may be related to the fact that upon entering cells, there may be physicochemical changes in the cell walls, which become damaged, causing porosity and ultimately leading to necrosis [78]. In other words, positive silver ions interact with the negatively charged cell membrane of bacteria through electrostatic attraction, increasing reactive oxygen species (ROS). Increased ROS destroys the cell wall structure [79]. AgNPs bind to the bacterial cell membrane, causing membrane damage, resulting in increased membrane permeability, and cell death due to leakage of intracellular components [80]. Several mechanisms have been proposed for the antibacterial activity of AgNPs, such as disruption of cell permeability and processes, penetration into bacterial cells, and damage through interactions with DNA and proteins [81, 82]. These results suggest that H-Hp/AgNPs have antibacterial activity.

#### 3.3.2. Antibiofilm Assay

**Figure 8:**
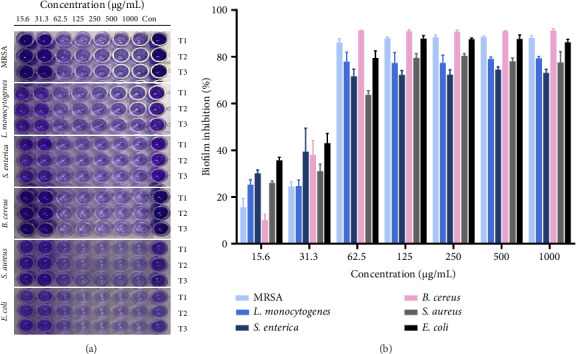
Antibacterial activity of different concentrations of H-Hp/AgNPs on different bacterial pathogens. Biofilm inhibition assay performed in a 96-well plate. T1, T2, and T3 indicated the experimental triplicate (a); and Biofilm inhibition percentage of H-Hp/AgNPs (b).

Biofilms are microbial aggregates encased in a matrix primarily composed of polysaccharides, which protect bacteria from the environment and play a key role in infections [83, 84]. These structures significantly increase antibiotic resistance, making pathogens up to 1000 times more resistant by blocking antibiotic penetration through extracellular polymeric substances (EPS) [85, 86]. Biofilms also enable bacteria to survive in harsh conditions, adhere to surfaces, and cause chronic infections [87]. Thus, targeting biofilms is crucial for combating antibiotic resistance and managing persistent infections.  Figure 8(a) shows the results of measuring the biofilm formation of *MRSA*, *L. monocytogenes*, *S. enterica*, *B. cereus*, *S. aureus*, and *E. coli* after treating various concentrations of samples (15.6, 31.3, 62.5, 125, 250, 500, and 1000 μg/mL) in a 96-well plate, and the experiment was performed in triplicate (T1, T2, and T3) for each bacteria. The dark purple color indicates that the biofilm content formed by dissolving the crystal violet accumulated in the biofilm is high. The results showed that all H-Hp/AgNPs showed a tendency for the inhibition rate to be at least twice that of the previous concentration at 62.5 μg/mL or higher (Figure 8(b)). In particular, H-Hp/AgNPs at 62.5 μg/mL or higher could inhibit biofilms by up to 80% in MRSA, and *B. cereus*, *E. coli*, and *B. cereus* showed the highest antibiofilm activity in all strains with an inhibition rate close to 90%. Overall, these biofilm inhibition results demonstrate that H-Hp/AgNPs can be used as practical antimicrobial agents. These results are consistent with previous studies in which AgNPs reduced and stabilized with *Lepidium sativum* seed mucilage showed the highest antibiofilm activity against *B. cereus* [88]. Previous reports have shown that AgNPs disrupt the protective layer by interfering with the proteins responsible for EPS synthesis. It is clear that they are effective in degrading EPS, killing bacterial colonies, and consequently reducing biofilms formed by gram-positive and gram-negative bacteria [89].

### 3.4. Anticancer

**Figure 9:**
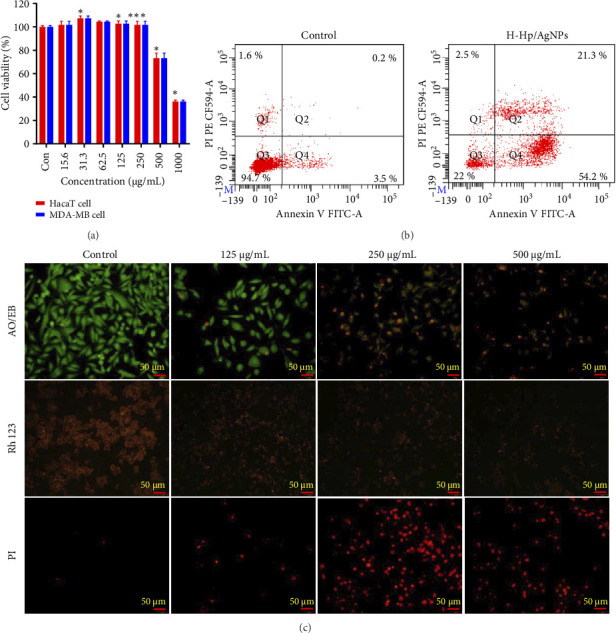
Anticancer activity of H-Hp/AgNPs. Cell viability of H-Hp/AgNP-treated HaCaT cells and MDA-MB cells (a); analysis of apoptosis of H-Hp/AgNPs on MDA-MB 231 cell line by flow cytometry (b); fluorescent staining analysis of H-Hp/AgNP-treated MDA-MB 231 cell. The different stages of apoptotic stages were observed by AO/EB, Rh123, and PI staining (c). The data are presented as the mean ± SD (*n* = 3), and difference evaluations are shown as ^∗^*p* < 0.05 and ^∗∗∗^*p* < 0.001.

The cell viability of H-Hp/AgNPs was evaluated in both normal (HaCaT) and cancer (MDA-MB 231) cell lines using WST assay (Figure 9(a)). The results showed that H-Hp/AgNPs induced a significant decrease in MDA-MB 231 cell viability in a concentration-dependent manner, demonstrating potent anticancer activity. At 500 and 1000 μg/mL, the viability of MDA-MB 231 cells was very low, 29.12% and 23.74%, respectively, which was significantly different from HaCaT cells, which showed cell viability of 73.15% and 36.13% (*p* < 0.01). In particular, at 250 μg/mL, the MDA-MB 231 cell viability was 3.3 times lower than that of HaCaT cells, showing a very significant difference in cytotoxicity (*p* < 0.001). The IC_50_ value of MDA-MB 231 cells was significantly lower than that of HaCaT cells, which further confirms the selective toxicity of H-Hp/AgNPs against cancer cells. The IC_50_ value of HaCaT cells was 835.7 ± 26.8 μg/mL, indicating relatively low cytotoxicity compared to the effect on cancer cells. Furthermore, H-Hp/AgNPs can target cancer cells at concentrations below 250 μg/mL without damaging normal cells. This cytotoxicity may be attributed to the ability of H-Hp/AgNPs to induce cell death by generating high levels of ROS in cancer cells [90]. Increased ROS production has been shown in various studies to be a cytotoxic anticancer mechanism of AgNPs and is likely to be one of the major pathways of cancer cell death. AgNPs have been reported to induce cytotoxicity in cancer cells by altering cell morphology, generating oxidative stress, and reducing cell viability in various cancer types [91].

Apoptosis is a form of programmed cell death, which is a natural process in the body [92]. It allows cells to die in a controlled and regulated manner, avoiding the harmful effects of uncontrolled cell death, like inflammation. This process plays a critical role in development, tissue homeostasis, and disease prevention by eliminating old, damaged, or unnecessary cells [93]. Apoptosis analysis using the Annexin V-FITC/PI assay was conducted to support the WST assay results, with a concentration of 250 μg/mL used for the analysis. This assay differentiates between apoptotic and necrotic cells: Annexin V-FITC binds to phosphatidylserine on the outer membrane of apoptotic cells, while PI binds to the nuclei of dead cells [94]. Cells stained with both Annexin V-FITC and PI are considered apoptotic, whereas cells stained only with PI are classified as necrotic, as their membranes are too compromised to allow Annexin V-FITC binding [95]. Annexin V-FITC/PI staining was performed using flow cytometry to distinguish between apoptotic and necrotic cells in MDA-MB 231 cells, with results shown in Figure 9(b). In the control group, 94.7% of the cells were viable, with early apoptosis detected in 3.5%, late apoptosis in 0.2%, and necrosis in 1.6% of the cells. In contrast, MDA-MB 231 cells treated with H-Hp/AgNPs exhibited significantly increased early and late apoptosis. Specifically, only 22% of the cells remained viable, with 54.2% showing early apoptosis, 21.3% showing late apoptosis, and 2.5% displaying necrosis. These results suggest that cancer cells can be eliminated in a more effective and controlled manner by effectively eliminating cancer cells through apoptosis rather than through unregulated necrosis that induces an inflammatory response [96]. Therefore, it can contribute to increasing the effectiveness of anticancer treatment and reducing side effects on normal tissues by minimizing abnormal inflammatory responses. These findings align with previous studies demonstrating that AgNPs' exposure reduces cancer cell viability [97].

We further validated the induction of apoptosis, mitochondrial membrane potential (MMP) loss, and apoptosis-associated nuclear changes, such as condensation and fragmentation, in MDA-MB 231 cells treated with H-Hp/AgNPs through fluorescence staining analysis (Figure 9(c)). AO/EB staining revealed significant differences compared to the control, with the number of red-stained apoptotic and necrotic cells increasing as the concentration of H-Hp/AgNPs increased. Notably, at 500 μg/mL, a reduction in stained cells was observed compared to 250 μg/mL, likely due to dead cells detaching during the washing process. MMP loss was visualized using Rh123 staining, which showed an increase in dark red-stained cells compared to the control group, confirming an increase in the number of cells with MMP loss. Similarly, PI staining indicated that as the concentration of H-Hp/AgNPs increased, the number of stained nuclei of dead cells also increased. As with AO/EB staining, a reduction in stained cells was observed at 500 μg/mL, likely due to cell detachment during washing, as seen at the lower concentration of 250 μg/mL.

### 3.5. Hemolytic Properties of H-Hp/AgNPs

**Figure 10:**
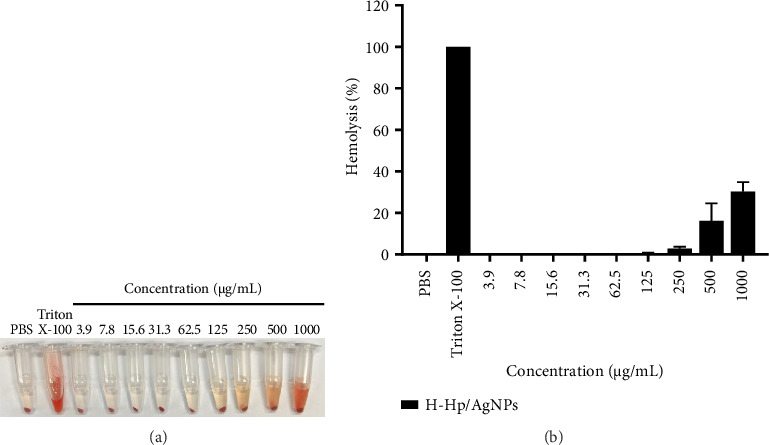
Photograph of hemolysis properties of H-Hp/AgNPs (a) and hemolysis percentage of H-Hp/AgNPs of the different concentrations (%) (b).

AgNPs are increasingly utilized in biomedical fields because of their potent antibacterial properties, and as a result, there is growing interest in their biocompatibility and potential applications in these areas [98]. However, AgNPs possess adverse effects on red blood cells, including membrane destruction, DNA damage, and the induction of congenital malformations [99]. Therefore, assessing the hemocompatibility of biosynthetic materials is crucial for practical biomedical applications, as interactions between materials and blood components can trigger cellular and humoral responses [100]. Various concentrations of H-Hp/AgNPs were incubated with red blood cells and centrifuged. The experimental results of H-Hp/AgNP-treated cells are shown in Figure 10(a). This was to determine the extent of red blood cell damage and the concentration of substances that cause hemoglobin release. The results show that increasing the concentration of H-Hp/AgNPs causes red blood cell damage. According to the American Society for Testing and Materials (ASTM F 756-00, 2000), substances that cause hemolysis above 5% are classified as toxic, those causing 2%–5% hemolysis are considered slightly hemolytic, and those causing less than 2% hemolysis are classified as nontoxic [101, 102]. In this study, the hemolysis of red blood cells was found to be 30.3% at 1000 μg/mL and 16.1% at 500 μg/mL, both concentrations classified as toxic. At a concentration of 250 μg/mL, hemolysis was 2.9%, indicating slight hemolysis, while at 125 μg/mL, hemolysis was minimal at 0.4%. Concentrations below 125 μg/mL showed 0% hemolysis and thus were classified as nontoxic with no hemolytic activity (Figure 10(b)).

## 4. Conclusion

The biosynthesized H-Hp/AgNPs were successfully characterized, and their biological activities were thoroughly investigated. These findings suggest that HME significantly enhances the extraction efficiency of bioactive substances, including astaxanthin. The UV–vis spectrum of H-Hp/AgNPs exhibited a prominent absorption peak at 420 nm, confirming successful nanoparticle formation. XRD analysis confirmed the crystallinity of AgNPs, and EDS confirmed the presence of silver. The zeta potential of −31.54 ± 0.2 mV, along with a PDI of 0.2 ± 0.3, indicated good stability and relative monodispersity. H-Hp/AgNPs exhibited enhanced antioxidant and antibacterial activities, particularly against B. cereus. Thus, H-Hp/AgNPs effectively inhibited the growth of *B. cereus*, demonstrating their potential as potent antibacterial agents. In addition, the nanoparticles exhibited antibacterial activity against various other bacterial strains, demonstrating their wide applicability. In particular, the antibacterial activity against MRSA suggests that they can contribute to addressing the challenges posed by multidrug-resistant bacteria. Biocompatibility tests revealed no toxicity to normal cells or red blood cells at concentrations below 500 μg/mL, while selective toxicity was observed against cancer cells. Apoptosis analysis further confirmed these results. This study suggests that AgNPs synthesized using HME-enhanced extraction methods could be effectively applied in pharmaceutical development.

## Figures and Tables

**Table 1:** Formulation ratio of Hp and biopolymers (%).

	N-Hp	H-Hp
Hp	100	70
HPCD	0	10
Lecithin	0	2.5
Ascorbyl palmitate	0	5
HPMC	0	10
Vitamin C	0	1.5
Vitamin E	0	1
Total	100	100

**Table 2:** Antibacterial activity of H-Hp and H-Hp/AgNPs.

Sample	Concentration (μg/mL)	ZOI (mm)					
		MRSA	*B. cereus*	*L. monocytogenes*	*S. aureus*	*E. coli*	*S. enterica*
H-Hp/AgNPs	250	6.2 ± 1	6.8 ± 1	8.5 ± 1.5	6.9 ± 0.1	8.6 ± 0.5	6
	500	6.7 ± 0.3	7.2 ± 1.6	9 ± 1	8.8 ± 0.3	9.6 ± 0.5	6
	1000	8.2 ± 0.3	10.2 ± 0.8	9.2 ± 0.8	9.3 ± 0.6	9.7 ± 0.6	9.8 ± 0.7

H-Hp	1000	—	—	—	—	—	—

TCH	1000	26.8 ± 0.3	22.5 ± 0.4	23.2 ± 0.3	23 ± 0.2	25.2 ± 0.3	27.5 ± 1

*Note:* The different concentration of H-Hp/AgNPs determined their ZOI against different bacterial pathogens.

**Table 3:** MIC and MBC of H-Hp/AgNPs.

	H-Hp		H-Hp/AgNPs	
	MIC (mg/mL)	MBC (mg/mL)	MIC (μg/mL)	MBC (μg/mL)
MRSA	50	—	62.5	125
*L. monocytogenes*	40	—	62.5	62.5
*S. enterica*	40	—	62.5	62.5
*B. cereus*	40	—	62.5	62.5
*E. coli*	50	—	125	250
*S. aureus*	50	—	250	250

## Data Availability

The data that support the findings of this study are available from the corresponding author upon reasonable request.

## Supporting Information

Supporting Informationa Additional supporting information can be found online in the Supporting Information section.

Figure S1: Antioxidant activity of ascorbic acid. ABTS (a); and DPPH (b). The data are presented as the mean ± SD (*n* = 3).
