# Supplementary material for: Bioactive Molecules of Microalgae Haematococcus pluvialis–Mediated Synthesized Silver Nanoparticles: Antioxidant, Antimicrobial, Antibiofilm, Hemolysis Assay, and Anticancer
Source: Bioinorg Chem Appl. 2025 May 2;2025:8876478. doi: 10.1155/bca/8876478 (PMC12064320; doi:10.1155/bca/8876478)
Supplement: Supporting Informationa — Additional supporting information can be found online in the Supporting Information section. [file 8876478.f1.docx]

**Bioactive Molecules of Microalgae *Haematococcus pluvialis*-Mediated Synthesized Silver Nanoparticles: Antioxidant, Antimicrobial, Antibiofilm, Hemolysis Assay, and Anticancer**

Yoo-Na Jeon^1^, Su-Ji Ryu^1^, Anbazhagan Sathiyaseelan^1^, Jong-Suep Baek^1,2*^

^1^ Department of Bio-Health Convergence, Kangwon National University, Chuncheon 24341, Republic of Korea.

^2^ BeNatureBioLab, Chuncheon 24206, Republic of Korea.

^*^Corresponding author

Tel: +82 33-257-5908, E-mail: [jsbaek@kangwon.ac.kr](mailto:jsbaek@kangwon.ac.kr)


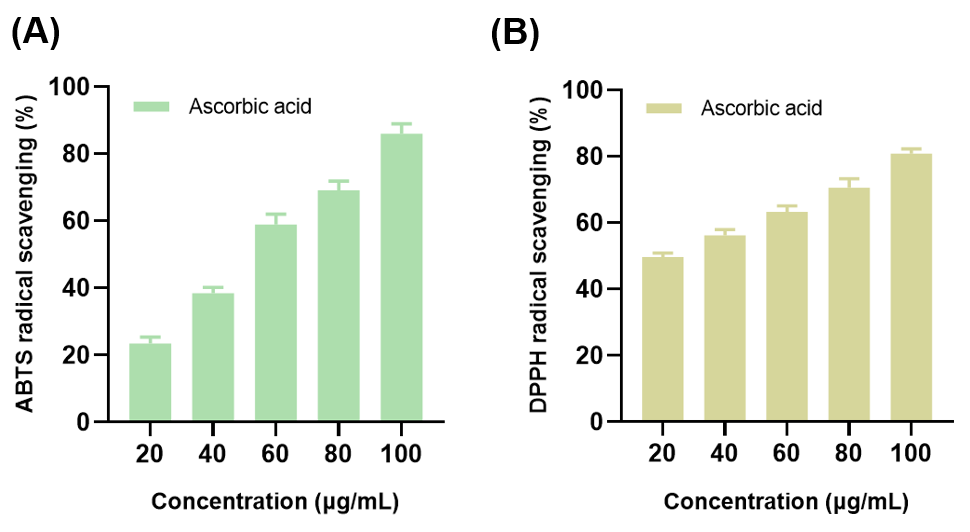


**Figure 1.** Antioxidant activity of Ascorbic acid. ABTS (A); and DPPH (B). The data are presented as the mean ± SD (n = 3).
